# Genomic Selection for Drought Tolerance Using Genome-Wide SNPs in Maize

**DOI:** 10.3389/fpls.2017.00550

**Published:** 2017-04-21

**Authors:** Mittal Shikha, Arora Kanika, Atmakuri Ramakrishna Rao, Mallana Gowdra Mallikarjuna, Hari Shanker Gupta, Thirunavukkarasu Nepolean

**Affiliations:** ^1^Division of Genetics, ICAR-Indian Agricultural Research InstituteNew Delhi, India; ^2^Centre for Agricultural Bioinformatics, ICAR-Indian Agricultural Statistics Research InstituteNew Delhi, India; ^3^Office of Director General, Borlaug Institute for South AsiaNew Delhi, India

**Keywords:** drought, genomic selection, transcription factor, SNP, parametric, non-parametric, semi-parametric

## Abstract

Traditional breeding strategies for selecting superior genotypes depending on phenotypic traits have proven to be of limited success, as this direct selection is hindered by low heritability, genetic interactions such as epistasis, environmental-genotype interactions, and polygenic effects. With the advent of new genomic tools, breeders have paved a way for selecting superior breeds. Genomic selection (GS) has emerged as one of the most important approaches for predicting genotype performance. Here, we tested the breeding values of 240 maize subtropical lines phenotyped for drought at different environments using 29,619 cured SNPs. Prediction accuracies of seven genomic selection models (ridge regression, LASSO, elastic net, random forest, reproducing kernel Hilbert space, Bayes A and Bayes B) were tested for their agronomic traits. Though prediction accuracies of Bayes B, Bayes A and RKHS were comparable, Bayes B outperformed the other models by predicting highest *Pearson correlation coefficient* in all three environments. From Bayes B, a set of the top 1053 significant SNPs with higher marker effects was selected across all datasets to validate the genes and QTLs. Out of these 1053 SNPs, 77 SNPs associated with 10 drought-responsive transcription factors. These transcription factors were associated with different physiological and molecular functions (stomatal closure, root development, hormonal signaling and photosynthesis). Of several models, Bayes B has been shown to have the highest level of prediction accuracy for our data sets. Our experiments also highlighted several SNPs based on their performance and relative importance to drought tolerance. The result of our experiments is important for the selection of superior genotypes and candidate genes for breeding drought-tolerant maize hybrids.

## Introduction

Traditional breeding strategies for selecting improved and resistant varieties of maize depending on the phenotypic trait have proven to be of limited success (Cushman and Bohnert, 2000). This direct selection is hindered by low heritability, and the existence of genetic interaction (e.g., epistasis), environmental-genotype interaction, and polygenic effects. This selection also takes a long period of time. Understanding the genetic basis of the plants' response to these various environments and the advent of new genomic technique and tools has allowed breeders to pave a way for selecting superior maize breeds (Tuberosa and Salvi, 2006).

Drought stress has the most detrimental effect on maize, leading to reduced yields in maize production (Nepolean et al., 2014). Several QTLs have been identified for drought tolerance in maize and those QTLs have been used to improve stress tolerance through marker-assisted breeding. However, marker-assisted breeding is limited to few major QTLs, thus minor QTLs are not part of the selection process, leading to a loss of genetic gain (Dekkers, 2004). To overcome this limitation, genomic selection (GS) has been proposed as a method to understand the effects of all the alleles across the genome to improve polygenic traits (Meuwissen et al., 2001). This method is advantageous over the traditional marker-assisted selection (MAS), as it addresses the effect of small genes which cannot be captured by the traditional MAS (Hayes et al., 2009).

GS is a form of MAS based on breeding values estimated from a genomic dataset that explores the genetic variances within each individual (Heffner et al., 2009). Current research in the area of genetic improvement explores GS as one of the approaches revolutionizing both animal and plant breeding (Hayes et al., 2009; Lorenzana and Bernardo, 2009). Genetic values of quantitative traits in maize and wheat datasets have been studied for the estimation of their higher predictive ability compared with molecular markers than pedigree information (Crossa et al., 2010).

GS reduced the selection time by almost half per cycle compared to the phenotypic selection for almost all traits in the different sets of maize, *Arabidopsis* and barley (Lorenzana and Bernardo, 2009). By replacing the phenotypic selection with the genomic estimated breeding value (GEBV), the gain for each unit cycle can be increased (Wong and Bernardo, 2008). GS can be appropriate even in the presence of modest molecular markers and diverse environmental conditions (Crossa et al., 2010). The prediction accuracy of breeding values in genomic selection has been found to be 0.58 for grain yield in maize (Zhao et al., 2012) and is estimated to be a better option than other methods considering the genetic gain each year (Lorenzana and Bernardo, 2009; Zhao et al., 2012).

Parametric (RR- Ridge Regression, LASSO- Least Absolute Shrinkage and Selection Operator, Elasticnet, Bayes A and Bayes B), semi parametric (RKHS- Reproducing Kernel Hilbert Space) and non-parametric (RF-Random Forest) models have been used to predict the genotype value, and machine learning programs (Long et al., 2007) have been proposed to develop prediction models for GS. These methods have been implemented in biparental (Lorenzana and Bernardo, 2009) and multi-parental populations (Heffner et al., 2011a) where the predictive ability using several models was compared among different datasets using *Arabidopsis*, wheat, maize, and barley.

Different genomic selection models have been examined in diverse panels of maize and wheat germplasm (De Los Campos et al., 2009; Crossa et al., 2010). GS contributed appreciable genetic gain for grain yield and stover quality in bi-parental maize population (Massman et al., 2013) and drought stress tolerance in tropical maize germplasm (Beyene et al., 2015). In maize, prediction accuracy of GS among the full-sibs was more accurate than unrelated crosses (Riedelsheimer et al., 2013). Among the GS models, rrBLUP and BSSV were found equally efficient in identifying the *Stenocarpella maydis* resistant maize inbred lines using DArTseq markers (Pedroso et al., 2016).

Little information exists in comparing the efficiency of genomic models to select the better genotypes for drought tolerance in subtropical maize germplasm. The objectives of the present investigation were to predict the GEBVs of genotypes under drought stress using seven GS models, to compare the prediction accuracies of those GS models, and to validate the top selected SNPs from GS models with the SNPs identified through previous GWAS experiment.

## Materials and methods

### Dataset

A set of 29619 cured SNPs, genotyped across a panel of 240 maize inbred lines from an earlier data set (Nepolean et al., 2013, 2014) was used in this experiment. The curation of dataset was done on the basis of MAF <0.05, heterozygosity > 5%, removal of “no calls” (SNPs not included in any cluster were categorized as “no calls”), monomorphs and unmapped SNPs. Briefly, the total genomic DNA from 240 genotypes was isolated with a Nucleopore DNA Sure Plant Mini Kit (Genetix Biotech Asia). SNP detection was performed using the Infinium HD Assay Ultra (Illumina, San Diego, CA, USA). SNP chips were hybridized with 50 ng × 4 μl DNA per sample. The Maize SNP50 BeadChip was used to scan the 240 samples with 24 samples per Sentrix Array Matrix (SAM). All 240 genotypes were genotyped with an Infinium Maize SNP50 BeadChip (Illumina, San Diego, California, USA) containing 56110 SNPs.

Phenotypic data under drought stress in three different environments (IARI, New Delhi: 28°N 77°E; 229m AMSL), ANGRAU, Hyderabad: 17°N 78°E; 536m AMSL and RRS, Karimnagar: 18°N 79°E; 264m AMSL) during post-rainy seasons of 2010/11 and 2011/12 generated earlier (Nepolean et al., 2014) were used for predicting the GEBV in the current experiment. The “mean data” obtained by pooling datasets of these 3 locations was also included in the present experiment. All the drought experiments was followed the alpha lattice design consists of 16 incomplete blocks, and each block comprised of 15 plots with 3 replicates. Drought trials were phenotyped for the anthesis-to-silking interval (ASI, in days), the grain yield (GY, kilograms per plot), the number of kernels per row (KR), the number of kernel rows (KRN), the ear girth (EG, in centimeters), the ear length (EL, in centimeters), and the 100-kernel weight (HKW, in grams). Mean data across location for each genotype was calculated using the restricted maximum likelihood (ReML) approach and the best linear unbiased predictors (BLUPs) was used for further analysis.

### Genomic selection models

Parametric models (RR, LASSO, EN, Bayes A, Bayes B), a non-parametric model (RF) and a semi-parametric model (RKHS) were used to estimate the genetic value of each genotype. Common variance was considered for all the markers by the ridge regression model (Meuwissen et al., 2001) and, therefore, for each marker effect it constricts uniformly. Estimation of RR βs was performed by minimizing the L_2_ panelized residual sum of squares (Riedelsheimer et al., 2012), where RR shrinks all marker effects toward zero rather than categorizing the markers as either significant or as having no effect (Breiman, 1995; Whittaker et al., 2000).

Another parametric model, LASSO, which estimates of the number of βs, was obtained by minimizing the residual sum of squares, and subjected to the constraint of L_1_-type penalty on regression coefficients (Technow et al., 2012). EN is a more generalized model that combines both the RR and LASSO penalties. EN's estimate of the number of βs was obtained by minimizing the residual sum of squares subjected to the constraints of both L_1_- and L_2_-type penalties on regression coefficients.

EN simplifies to RR when α = 1 and to LASSO when α = 0. For any other value of α (0 < α <1), EN is used. The L_1_ part of EN performs automatic variable selection while L_2_executes grouped selection and stabilizes the solution paths with respect to random sampling. Predictive accuracies and significant SNPs for all traits were estimated through RR, LASSO, and EN with the use of an R package “glmnet” with penalty parameters optimized *via* ten-fold cross validation (Friedman et al., 2010). The significant SNPs estimated on the basis of variable importance were compared with the previous genome-wide association (GWA) results from the water-stressed maize panels (Nepolean et al., 2014).

The other two parametric models, Bayes A and Bayes B (Meuwissen et al., 2001) do not consider the common variance across the effects of SNPs. The Gibbs sampler for 50,000 repetitions fitting the model was computed by discarding the first 5,000 samples as a burn-in and saving one of each of the ten samples for computing the posterior means for parameters. The Bayes A method assumes conditional distribution of each marker effect (given its variance) to follow a normal distribution. If the π value becomes zero, then the Bayes B model shrinks to Bayes A. We used the “BGLR” R package for the implementation of both Bayes A and Bayes B (De Los Campos et al., 2013).

A kernel function is used by the RKHS method to translate datasets of markers into a square matrix to be used in a linear model. There is a possibility that this method might capture non-additive genetic effects because of its ability to perform non-linear regression in a higher dimensional space. RKHS prediction was performed using “BGLR” (Bernardo and Yu, 2007). The model can be formulated as follows:

(1) $$Y=W\mu +K_{h}\alpha +\;\varepsilon$$

where ε can be defined as a vector of random residuals and μ as a vector of fixed effects. The parameters α and ε have independent distributions. The matrix *K*_*h*_ depends on a kernel function with the smoothing parameter *h*, which measures the “genomic distance” between genotypes and can be interpreted as a correlation matrix. The “genomic distance” between genotypes is measured by *K*_*h*_, where h represents the smoothing parameter and can be elucidated as a correlation matrix. Here, the Gaussian kernel was used on the genetic distance. The decay rate of correlation between genotypes is regulated by the *h* parameter.

Genomic prediction using parametric and semi-parametric models RR, LASSO, EN, Bayes A, Bayes B, and RKHS was based on 29,619 SNPs, while prediction using a non-parametric model Random Forest (RF) was performed using 5420 SNPs that were randomly selected from a set of 29,619 SNPs. The RF test uses a random subset of predictors to form a collection of regression trees on the basis of bootstrap samples of observations. This model was implemented using the R package “RandomForest” (Liaw and Wiener, 2002) where the number of trees was adjusted to 1,000, keeping *mtry* at *p/3*. Prediction accuracy for all agronomic traits was calculated through “Pearson correlation coefficient” between observed and predicted value in all seven GS models.

### Validation

To compute predictive accuracies, a 10-fold cross-validation (CV) scheme was applied and iterated 10 times. In each iteration, 10 disjoint subsets of genotypes were formed randomly where one random subset was used as a validation set and the other nine subsets were used as a training population to estimate the parameters of the model used for prediction of excluded genotypes in the validation set. The Pearson correlation between observed and predicted values was calculated in each round. This procedure was iterated 10 times to obtain 100 cross-validation runs. The predictive ability was calculated as the Pearson correlation coefficient between observations and cross-validated GEBVs were thus referred for accuracy.

## Results

### Mean performance

Under drought condition, the performance of 7 agronomic traits—ASI, GY, KR, KRN, EG, EL, and HKW were recorded in all three locations. In summary, the mean data from all three locations explained that ASI under drought varied from 2 to 12 days with a mean value of 6 days and standard deviation of 2.53. GY had a range of 0.2–2.2 with an average of 1.7 and a standard deviation 1.92. For KRN, the mean and standard deviation was 31 and 2.57, respectively. Other agronomic traits i.e., EL, EG, KR, and HKW, the range varied from 7.8–17.5, 1.8–4.1, 10.9–18 and 15–32 with an average of 13.4, 3.3, 13, and 26 respectively (Nepolean et al., 2014).

### Prediction accuracy of GS models

Accuracy of the seven GS models was predicted for all seven traits phenotyped for drought stress at the three locations (Table 1). While comparing the prediction accuracies among these traits and locations, we observed that the highest prediction accuracies of 0.93, 0.91, and 0.92 were identified for ASI, EG, and HKW, respectively, in Karimnagar, whereas for GY, Hyderabad and Karimnagar provided the best results, and Karimnagar and IARI showed better results than Hyderabad for EL. The highest prediction accuracy for KRN was identified in Hyderabad with a value of 0.91. KR was the only trait in which all the 3 locations provided consistent results. Across all traits, the maximum prediction accuracy was found for ASI and the minimum for KR. It was examined that among the 3 locations, Karimnagar provided the best results.

**Table 1:** **Prediction accuracies of agronomic traits predicted by seven GS models under drought stress in subtropical maize**.

**Traits**	**GS Models**						
	**RR**	**LASSO**	**EN**	**RF**	**RKHS**	**Bayes A**	**Bayes B**
**LOCATION: IARI, NEW DELHI**							
ASI	0.82	0.77	0.77	0.83	0.91	0.90	0.92
EG	0.69	0.71	0.71	0.85	0.89	0.87	0.89
EL	0.62	0.61	0.61	0.86	0.89	0.90	0.91
GY	0.60	0.53	0.63	0.84	0.89	0.88	0.87
HKW	0.86	0.84	0.83	0.85	0.90	0.88	0.90
KR	0.77	0.71	0.76	0.86	0.90	0.87	0.89
KRN	0.71	0.69	0.72	0.86	0.89	0.88	0.90
**LOCATION: HYDERABAD**							
ASI	0.28	0.28	0.28	0.86	0.91	0.91	0.90
EG	0.78	0.78	0.75	0.85	0.89	0.88	0.90
EL	0.72	0.71	0.72	0.85	0.89	0.90	0.89
GY	0.64	0.63	0.61	0.83	0.89	0.90	0.89
HKW	0.69	0.70	0.68	0.89	0.89	0.88	0.90
KR	0.72	0.73	0.72	0.86	0.90	0.88	0.90
KRN	0.77	0.66	0.84	0.86	0.90	0.89	0.91
**LOCATION: KARIMNAGAR**							
ASI	0.30	0.30	0.30	0.86	0.91	0.93	0.92
EG	0.78	0.70	0.79	0.87	0.90	0.89	0.91
EL	0.73	0.65	0.70	0.86	0.90	0.87	0.91
GY	0.56	0.56	0.61	0.85	0.89	0.89	0.90
HKW	0.66	0.69	0.71	0.88	0.89	0.89	0.92
KR	0.77	0.71	0.74	0.86	0.89	0.89	0.90
KRN	0.42	0.40	0.35	0.85	0.90	0.89	0.90
**MEAN**							
ASI	0.91	0.92	0.92	0.84	0.98	0.97	0.97
EG	0.88	0.90	0.87	0.84	0.98	0.97	0.96
EL	0.90	0.90	0.88	0.84	0.99	0.97	0.97
GY	0.78	0.81	0.79	0.80	0.98	0.93	0.95
HKW	0.91	0.93	0.92	0.86	0.99	0.97	0.97
KR	0.91	0.93	0.90	0.86	0.98	0.96	0.96
KRN	0.94	0.92	0.91	0.85	0.98	0.95	0.96

Prediction accuracy and standard deviations ranged between 0.28–0.92 and 0.03–0.06 (Hyderabad), 0.53–0.92 and 0.02–0.06 (IARI), and 0.30–0.93 and 0.02–0.06 (Karimnagar), respectively, across all traits and models. RKHS, Bayes A, and Bayes B showed no difference in the prediction accuracy above 0.04, while RR, LASSO and EN showed prediction accuracies above 0.03 for all traits, except for KRN, which showed a difference of more than 0.07 at all locations. Bayes B estimated the highest prediction accuracy for all traits except for EG and KRN. Bayes A and RKHS provided the second best prediction accuracy with a drop of 0.01–0.02 (Hyderabad); 0.01–0.03 (IARI), except for GY; and 0.01–0.04 (Karimnagar), except for ASI, respectively, compared to Bayes B.

A great fall in the prediction accuracies for certain traits (ASI, GY, and KRN) under specific models over different locations was observed. Ridge, lasso and EN models predicted ASI with less accuracy (0.28) in Hyderabad. Similarly, in IARI, less prediction accuracy was found for GY (0.6), whereas Karimnagar, ASI (0.3), GY (0.56), and KRN (0.4) had less accuracy compared to the other traits and locations. For GY, the best results (0.9) for Hyderabad and Karimnagar were predicted by both Bayes A and Bayes B, while for IARI, RKHS predicted the best value (0.89). Overall, the prediction accuracy of the mean location was better than that for individual locations.

In location-wise comparison, it was noticed that though the results of RKHS, Bayes A and Bayes B were quite similar but the highest prediction accuracy was obtained from the Bayes B model in all 3 locations namely Hyderabad, IARI and Karimnagar while in the mean dataset RKHS was slightly better over the Bayes B.

### SNPs identified through different models

**Figure 1:**
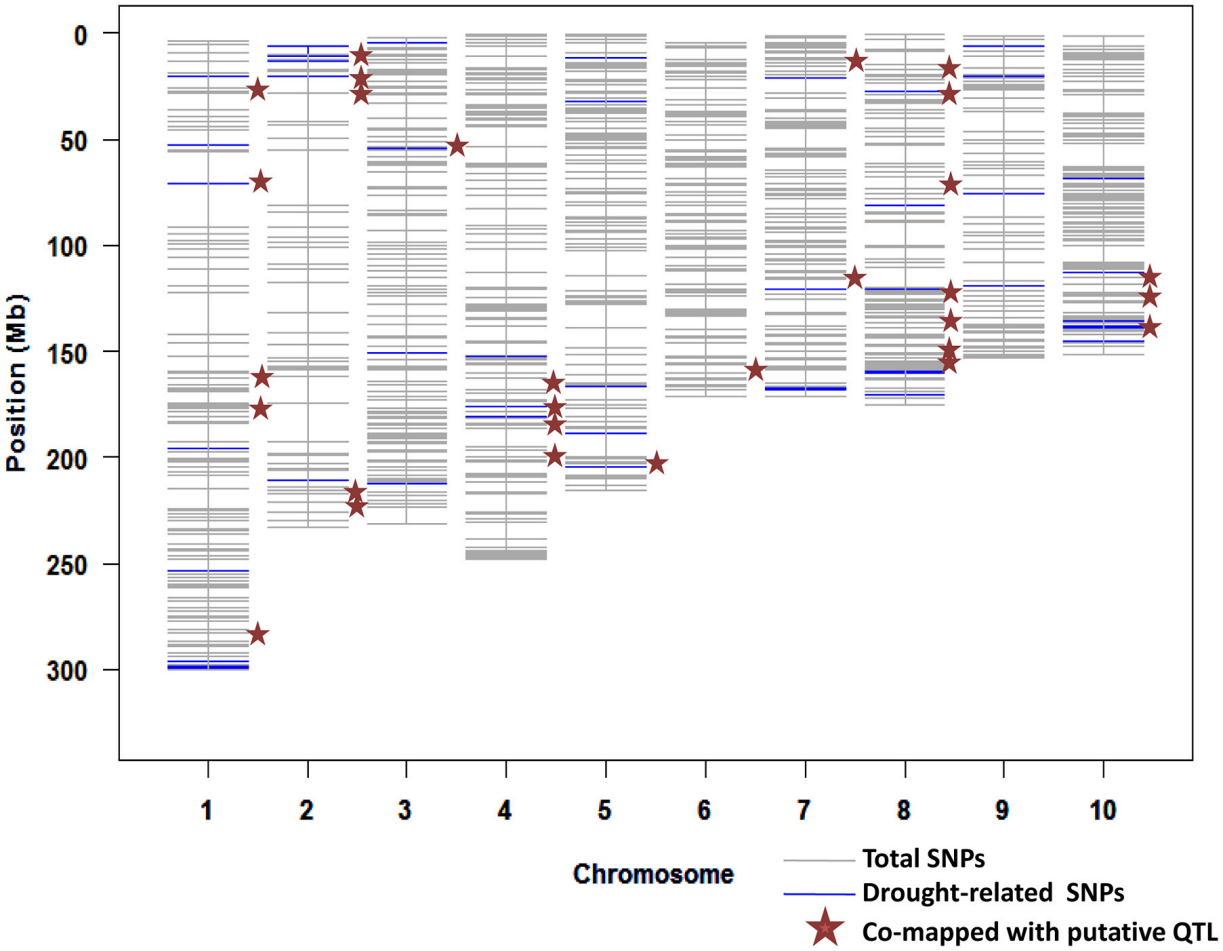
**Distribution of SNPs with higher marker effects mapped for different traits and locations from the Bayes B model; several SNPs were associated with drought-related transcription factors and co-mapped with putative QTLs**.

We had estimated the prediction accuracy for seven GS models. From these models, the Bayes B model provided the maximum accuracy for six of the seven traits across several environments. A set of the top 100 SNPs with the highest marker effect observed in each trait and environment was selected using the Bayes B since it produced highest accuracy in all three locations. From this exercise, a total of 2800 SNPs with the highest marker effect across several datasets (traits + environments) were identified. Out of these SNPs, 1053 SNPs were unique (Supplementary Table S1). These SNPs distributed across the genome, ranging from 52 SNPs in chromosome 2–150 SNPs in chromosome 1 (Figure 1).

Out of the 1053 SNPs, a set of 77 consistent SNPs identified across several traits and locations (Table 2) were selected as a test for understanding their functional relationships with drought-tolerant genes. The maize gene models explained that these 77 SNPs distributed across the genome were mapped 10 drought-responsive TFs within their 150 Kb region. *CAMTA* mapped within 41 Kb region followed by *bHLH* (73 Kb), *bZIP* (92 Kb), *NF-YB* (101 Kb), *NF-YA* (108 Kb), *GRAS* (120 Kb), *WRKY* (125 Kb), *AP2-ERF* (148 Kb), *MYB* (149 Kb), and *NAC* (149 Kb). The *AP2-ERF* TF family was mapped close to the maximum number of SNPs (30) on all chromosomes; whereas chromosome 9 had the most drought-responsive SNPs (6) and chromosome 3 contributed only one drought-responsive SNP. The *MYB* TF family was mapped to 17 SNPs located on all chromosomes except on chromosomes 1 and 10. The SNP PZE-109076471 on chromosome 9 was mapped 2 Kb from *MYB* TF. Both *WRKY* and *GRAS* TFs mapped 10 unique and seven SNPs in their vicinity, respectively. The *BHLH* TF family encompassed five SNPs on different chromosomes, including an SNP (PZE-110088632) on chromosome 10, which was located only 732 bp away from the *BHLH* TF. The *NF-YA* (3), *bZIP* (2), *NAC* (1), *CAMTA* (1), and *NF-YB* (1) TFs mapped 3, 2, 1, and 1 SNPs, respectively. In addition, SNPs were also mapped close to more than one TF family. The SNP SYN38859 on chromosome 2 was mapped close to *MYB* and *AP2-ERF* at a distance of 2 and 116 Kb away, respectively, and another SNP (PZE-107128846) on chromosome 7 was mapped at a distance of 54 and 108 Kb away from the *GRAS* and *NF-YA* TFs, respectively.

**Table 2:** **A set of 77 consistent SNPs identified through Bayes B mapped drought-responsive transcription factors within the 150 Kb region**.

**SNP**	**Gene model**	**Chr**	**SNP Position**	**Gene Start**	**Gene End**	**Annotation**
PZE-101127875	GRMZM2G039112	1	162280117	162426395	162428231	EREB168
PZE-101152541	GRMZM2G309731	1	195764754	195878179	195878689	EREB119
SYN28647	GRMZM2G003466	1	20132877	20094963	20096296	EREB101
SYN2521	GRMZM2G144744	1	266030836	266094769	266097836	GRAS8
SYN32645	GRMZM2G110067	1	71014176	71022756	71024504	GRAS27
PZE-101135368	GRMZM2G008250	1	174834248	174845979	174849344	NF-YA2
SYN122	GRMZM2G030272	1	52861261	52919938	52921358	WRKY32
PZE-101205664	GRMZM2G070211	1	253475788	253502280	253505400	WRKY102
SYN37966	GRMZM2G068967	2	10684223	10786158	10786914	EREB97
SYN38859	GRMZM2G028969	2	20125223	20096078	20097002	EREB185
SYN29038	GRMZM2G475678	2	20642295	20563651	20564721	EREB61
SYNGENTA13688	GRMZM2G174917	2	5693114	5562976	5564647	EREB47
SYN6387	GRMZM2G038722	2	13208848	13298750	13300526	MYB13
SYN38859	GRMZM2G105137	2	20125223	20122023	20123440	MYB104
SYN33932	GRMZM2G040349	2	210814330	210788762	210792895	NF-YA3
SYN456	GRMZM2G071907	2	11850510	11753531	11755126	WRKY50
SYN36398	GRMZM2G117851	3	212101837	212179339	212194812	bZIP99
PZE-103120110	GRMZM2G060216	3	176807786	176800215	176808889	bZIP11
PZE-103008756	GRMZM2G133168	3	4686561	4660985	4665930	EREB103
PZE-103093412	GRMZM2G082387	3	150730803	150830511	150832666	GRAS4
SYN31097	GRMZM2G051256	3	54498010	54469147	54472943	MYB40
PZE-103149619	GRMZM2G167829	3	201971540	201931089	201932734	MYB151
PZE-104099837	GRMZM2G018398	4	175944355	176036953	176039523	EREB14
PZE-104078796	GRMZM2G072926	4	152168462	152162187	152163244	EREB176
PZE-104105965	GRMZM2G029323	4	181089587	181057725	181059564	EREB17
PZE-104000308	GRMZM2G018254	4	609379	513060	515231	GRAS48
PZE-104079825	GRMZM2G098800	4	153293870	153196436	153199541	GRAS80
PZE-104115471	GRMZM2G017268	4	196944239	197071819	197073073	MYB63
PZE-104028583	GRMZM2G157306	4	34490747	34467629	34474170	MYBR92
PZE-104108817	GRMZM2G063216	4	184552967	184639458	184643071	WRKY16
PZE-105044893	GRMZM2G024871	5	31776003	31857938	31858460	EREB74
PZE-105109854	GRMZM2G016434	5	166412867	166319262	166321982	EREB129
PZE-105169336	GRMZM2G021369	5	210395726	210273738	210275023	EREB136
SYN908	GRMZM2G024973	5	11663525	11781976	11784448	GRAS44
SYN14867	GRMZM2G161512	5	41641428	41686328	41688450	MYB150
PZE-105064380	GRMZM2G145041	5	64074058	64117509	64120689	MYBR96
PZE-105133279	GRMZM2G170049	5	188960849	188916348	188920991	MYB26
PZE-105031680	GRMZM2G095239	5	17158383	17104261	17116833	MYBR27
PZE-105156453	GRMZM2G011789	5	204435442	204332560	204333737	NF-YB6
SYN4309	GRMZM5G846057	6	165411234	165487861	165489392	EREB34
PZE-106026281	GRMZM2G380377	6	62617003	62553730	62555335	EREB56
PZE-106018957	GRMZM2G089636	6	38316669	38422614	38424509	GRAS60
PZE-106065562	GRMZM2G048910	6	117930951	117954549	117956404	MYB106
SYN28345	GRMZM2G171569	7	21261412	21149818	21151267	EREB64
PZE-107069244	GRMZM2G052667	7	120468208	120351650	120354724	EREB102
PZE-107128846	GRMZM2G169636	7	165104257	165156675	165158312	GRAS81
PZE-107135434	GRMZM2G150841	7	168141869	168289381	168291477	MYB23
SYN4566	GRMZM2G056407	7	167528708	167607978	167609967	MYB94
PZE-107101710	GRMZM2G172327	7	150099406	150087003	150088438	MYB14
PZE-107128846	GRMZM2G038303	7	165104257	164991494	164995805	NF-YA3
PZE-108077632	GRMZM2G700665	8	131963205	132044001	132047428	EREB110
PZE-108069655	GRMZM2G174347	8	121038497	120960110	120961302	EREB92
SYN4110	GRMZM2G044077	8	27006540	26881888	26883485	EREB96
PZE-108048529	GRMZM2G120401	8	81105160	80979479	80980774	EREB194
PZE-108106293	GRMZM2G129154	8	159110821	159017496	159019119	GRAS2
SYN17469	GRMZM2G136887	8	140339489	140378673	140386322	MYBR101
PZE-108108473	GRMZM2G134073	8	160576714	160424732	160426914	NAC9
PZE-108127850	GRMZM2G029292	8	170252297	170359322	170386361	WRKY35
PZE-109019740	GRMZM2G073982	9	20041676	20112432	20114447	EREB33
PZE-109016273	GRMZM2G301860	9	16227362	16252231	16253405	EREB122
PZE-109046027	GRMZM2G073047	9	75456354	75472705	75473919	EREB39
PZE-109005418	GRMZM5G852704	9	5954786	5876876	5877925	EREB31
PZE-109019829	GRMZM2G073982	9	20238182	20112432	20114447	EREB33
PZE-109016446	GRMZM2G301860	9	16395239	16252231	16253405	EREB122
PZE-109076471	GRMZM2G098179	9	119314448	119310947	119312449	MYB52
PZE-109076511	GRMZM2G098179	9	119414197	119310947	119312449	MYB52
PZE-110057129	GRMZM2G152661	10	109538261	109572710	109580177	CAMTA5
PZE-110058576	GRMZM2G023708	10	112403513	112305177	112306206	EREB125
PZE-110102744	GRMZM2G076602	10	145455965	145350317	145352828	EREB212
PZE-110083667	GRMZM2G173429	10	135747046	135800932	135802925	GRAS22
PZE-110036061	GRMZM2G090594	10	68581754	68725531	68726795	WRKY67
PZE-110068347	GRMZM2G031963	10	124790354	124659347	124664396	WRKY59

The set of 1053 SNPs detected using the Bayes B model were matched with the previously identified 67 significant SNPs from GWAS models of GenABEL and GAPIT (Nepolean et al., 2014). We found 10 SNPs which were commonly identified by GS as well as GWAS models (Table 3). These SNPs were mapped on different chromosomes i.e., chromosome 1 (7 SNPs), chromosome 3 (1 SNP), chromosome 4 (1 SNP), and chromosome 10 (1 SNP). All these SNPs were associated with 13 maize gene models and had drought-related functions. Six SNPs were annotated as transcription factors including *MYB, bHLH, NF-YA*, and *FAR1* while rest of them as *chaperone protein dnaj 49-like, duf231 domain containing family protein, tubulin beta-1 chain, glutathione peroxidase*, and *NADP-malic enzyme*.

**Table 3:** **High marker effect SNPs from the Bayes B GS model matching with the previous GWAS models**.

**Common SNPs in GS and GWAS**	**Chr**	**Position (in bp)**	**Gene model**	**Annotation**	**Drought-related function**
PZE-101100942	1	96540960	AC197099.3_FGT005	MYB-related (TF)	Stomatal regulation
PZE-101125101	1	157957977	GRMZM2G418217	Protein far1-related sequence 5-like	ABA-signaling
PZE-101130083	1	166240443	GRMZM2G570020	bHLH (TF)	Stomatal regulation
PZE-101130084	1	166240542	GRMZM2G570020	bHLH (TF)	Stomatal regulation
PZE-101130213	1	166556661	GRMZM2G071385	chaperone protein dnaj 49-like	Homeostasis
PZE-101130292	1	166625734	GRMZM2G038855	duf231 domain containing family protein	Water uptake
PZE-101135368	1	174834248	GRMZM2G008250	Nuclear transcription factor y subunit a-2	Stomatal regulation
PZE-103046076	3	47639590	GRMZM2G133802	Tubulin beta-1 chain	Root development
PZE-104061181	4	119441233	GRMZM2G009275	tpa: hlh dna-binding domain superfamily protein	Stomatal regulation
SYNGENTA14972	10	138496646	GRMZM5G822829	bhlh domain protein	Stomatal regulation
SYNGENTA14972	10	138496646	AF466202.2_FGP007	tpa: rna recognition motif containing family protein	Plant growth and development under drought
SYNGENTA14972	10	138496646	GRMZM5G884600	glutathione peroxidase	ROS homeostasis
SYNGENTA14972	10	138496646	AF466202.2_FGP001	NADP-malic enzyme	Ion homeostasis

## Discussion

Many studies have implemented GS to test the gains in various genetic enhancement programs (Bernardo and Yu, 2007; Wong and Bernardo, 2008; Mayor and Bernardo, 2009; Shengqiang et al., 2009). A high level of correlation between true breeding values and the GEBV is found to be sufficient for genomic selection based on marker data (Heffner et al., 2009).

Different approaches have been used to determine breeding values from GS models–penalized regressions (RR, LASSO, and EN), Bayesian approaches (Bayes A and Bayes B), and non-linear regressions (RKHS and RF) (Hayes et al., 2009; Heslot et al., 2012; Nepolean et al., 2013). Non-linear regression models are studied for higher prediction accuracy over penalized models. In our study, we found higher prediction accuracy for both non-linear models compared to penalized models, with a maximum difference of 0.25 between non-linear models and penalized models across all seven traits. Among several different GS models, a better accuracy is found for non-linear models since they can capture non-additive genetic effects (Technow et al., 2012). However, if the additive genetic effects are solely included, using nonparametric models may not yield the expected level of accuracy.

The least prediction accuracy was observed for regularized linear models in this study. This model can be supported by the presence of epistatic interactions which may lower the performance of linear models (Ogutu et al., 2012). Among the penalized models, we observed better prediction accuracy for EN and RR than for LASSO. These results were in agreement with a previous study where EN outperformed LASSO in terms of consistency of model selection and prediction accuracy (Zou and Hastie, 2005).

Bayes B is a variable selection operator, and identifies a subset of markers with larger effects particularly those controlled by a few large QTLs. In our study, the Bayes B approach provided better prediction accuracy across all data (location + trait) compared to other GS models. Previous studies have also reported the better performance of Bayes B as compared to other GS models (VanRaden et al., 2009; Daetwyler et al., 2010; Jannink et al., 2010). We also observed that RKHS showed the highest prediction accuracy but was restricted to a few datasets. The variation in prediction superiority for RKHS has also been observed in previous results (Shengqiang et al., 2009; Crossa et al., 2010). The Bayesian model incorporates additive genetic effects, while RKHS captures complex epistatic interactions (Gianola and Van Kaam, 2008). Therefore, one would expect the Bayesian method to perform well in traits where additive effects play a central role and RKHS to perform well in traits where epitasis is more relevant. This also implied that both additive and non-additive components play significant role in trait expression in variable magnitude depending upon the genetic architecture of the traits (Crossa et al., 2010).

Our results showed the presence of variation in predicting the breeding values in different locations which explained that breeding values are shaped up by the environment. The results were also in coherent with our previous GWAS results (Nepolean et al., 2014) where location-specific SNPs were identified. It is also interesting to note that several SNPs were consistent in across locations as well as across traits in the GWAS study.

Genotype × Environment (G × E) interaction is an important component of genetic variability (Crossa et al., 2010, 2011). Various genomic selection studies have included G × E effect while predicting the values in across environments (Heffner et al., 2011b; Resende et al., 2011), within environments or group of environments (Burgueño et al., 2012; Dawson et al., 2013; Ly et al., 2013; Heslot et al., 2014) or using marker-by-environment predictions (Jarquín et al., 2014; Lopez-Cruz et al., 2015).

In our experiment, the Bayesian models out-performed the RR and LASSO models, and this result may be because Bayesian models utilize marker-specific shrinkage of effects, while RR and LASSO equally penalize entire marker effects (Meuwissen et al., 2001). This effect was evident in this study where Bayesian models out-performed the BLUP model by a difference of 0.25 in prediction accuracies. The latter model considers equal variance in all markers, and does not require preliminary information on the variance of marker effects. However, this information is required in Bayesian approaches to estimate prediction accuracies. In addition, RR also incorporates familial relationships and is hence inferior to Bayesian method (Habier et al., 2007). Since the Bayes B method estimates higher prediction accuracies for six of seven traits, it was selected for further validation of SNPs associated with drought tolerance.

### Functional mechanisms of selected top SNPs

The SNPs selected based on their marker effects were found to be associated with 10 droughts responsive TFs. The collective role and the interaction of those SNPs with various stress-related mechanisms at a functional level are discussed below.

#### Hormone signaling

Abscisic acid (ABA) is a phytohormone stimulated in response to developmental and environmental stimuli. Early stages in ABA signaling involve ABA receptors, *phosphatases*, and *kinases* that control the regulation of their targets (Soon et al., 2012). The binding of ABA molecules to their receptors stimulates the inhibition of *proteinphosphatases* (*PP2Cs*), which, in turn activates *SNF1-related protein kinase 2* (*SnRK2*) (Ng et al., 2011; Soon et al., 2012). *SnRK2* is an important signaling molecule that phosphorylates its downstream targets, including the transcription factors *NAC, bZIP, HSF, MYB, WRKY*, and *RAV1* (belonging to *AP2-ERF* family; Furihata et al., 2006; Fujita et al., 2009; Kim et al., 2012; Feng et al., 2014). ABA-inducible *bZIP* transcription factors containing ABA-responsive elements (ABRE) regulate HSFs in a drought-responsive manner (Yoshida et al., 2010; Bechtold et al., 2013).

**Figure 2:**
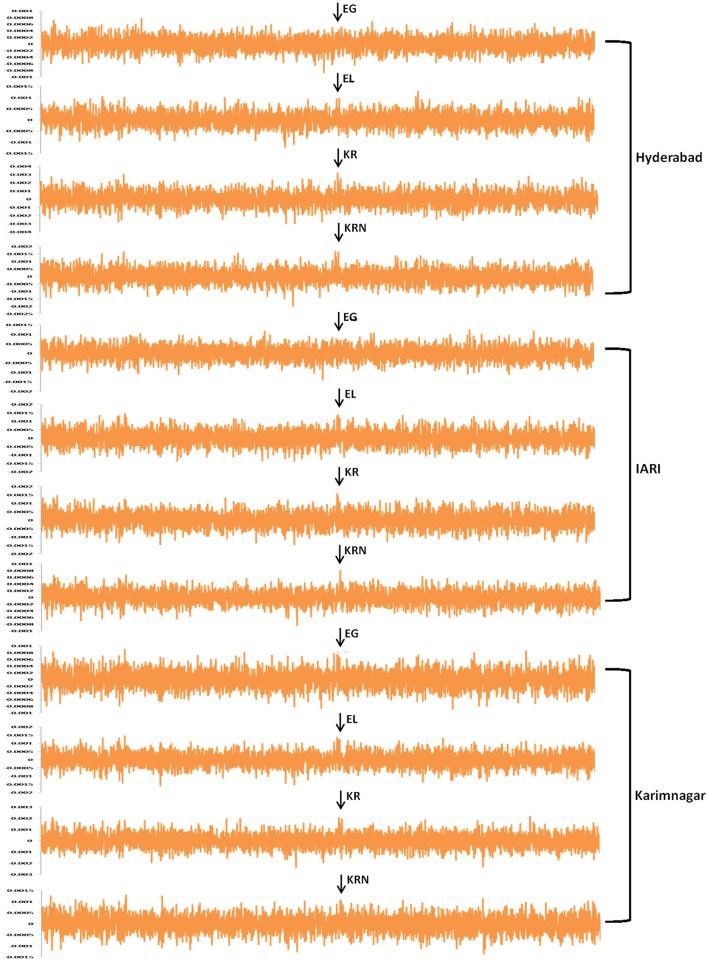
**The marker effect of a consistent SNP PZE-104079825 associated with the ROS scavenging GRAS transcription factor mapped on chromosome 4 from various traits and locations**.

Under drought stress, ABA is accumulated in guard cells where the closing of stomata is dependent upon H_2_O_2_ synthesis produced in the ABA-signaling pathway (Bright et al., 2006). Drought-inducible transcription factors *WRKY* (Ren et al., 2010), *NF-YA* (Gao et al., 2015), *MYB* (Seo and Park, 2009), *CAMTA* (Pei et al., 2000; Chen et al., 2004; Pandey et al., 2013), *C2H2* (Huang et al., 2009) and *bHLH* (Abe et al., 2003; Seo et al., 2011) trigger stomatal closure under the effect of ABA alone in drought stress. *ERF* is another drought-responsive transcription factor stimulated under the effect of ABA but is also integrated with other two hormones—jasmonic acid and ethylene—which induce the closing of stomata (Cheng et al., 2013). The *ERF* transcription factor mapped close to three SNPs, distributed on chromosomes 5, 7, and 8 was co-localized with quantitative trait loci (QTLs) mapped for EL, GY, ASI, and KRN in earlier studies (Guo et al., 2008; Messmer et al., 2009; Nikolić et al., 2012; Figure 2).

Gibberellic acid is another plant hormone that promotes growth and cellular elongation; however, its impact on drought stress is not completely understood. GA-responsive transcription factors such as *SCARECROW* (belonging to *GRAS* family) are studied for their involvement in cellular differentiation in the *Arabidopsis* root meristem (Sabatini et al., 2003; Ma et al., 2010). These plant hormones constitute a signaling network that involves various receptors, phosphatases, kinases, calcium-binding signaling molecules, and transcription factors.

#### Photosynthesis

Maintenance of photosynthesis is an adaptive trait that contributes to an improvement in grain yield in drought stressed plants. The transgenic maize with an increased *NF-YB* activity is studied for drought tolerance with a maintained photosynthetic rate and high yield (Nelson et al., 2007). These plants also showed good stomatal conductance and high chlorophyll index in water stressed conditions. In addition, the *NF-YB* transcription factor mapped close to an SNP on chromosome 5 and was co-localized with a QTL for GY, which was previously identified by Nikolić et al. (2012). Another transcription factor *CAMTA* is identified as a regulator of the photosynthetic machinery, where the T-DNA insertion line of *AtCAMTAs* has low photo system II efficiency under drought stress (Pandey et al., 2013).

#### Root development

A deeper root system is capable of accessing all soil moisture in a drought-stressed plant system. Root development due to drought tolerance has been observed in *SNAC1*-overexpressing transgenic cotton plants (Liu et al., 2014), and root enlargement has been observed in root-overexpressing *OsNAC10* drought-stressed rice plants (Jeong et al., 2010). However, the inhibition of lateral root development is an adaptive response to drought stress (Xiong et al., 2006), whereas reduced lateral root formation and improved drought tolerance have been found in *Arabidopsis MYB96*-overexpressing mutant plants (Seo and Park, 2009).

## Conclusion

Our results showed that Bayes B is superior to the other GS models in predicting the genomic values of the studied genotypes. Using Bayes B, we found 77 SNPs that are significant by their marker effects and are related to drought-responsive TFs. We also identified common SNPs from current GS model and previous GWAS models. These significant SNPs are related to many functions, such as stomatal closure, root development, hormonal signaling and photosynthesis. As these SNPs are drought-related and involved in various molecular functions, they can be further used for the development of drought-tolerant hybrids.

## Availability of supporting data

The raw SNP data (Submission No. 10.6070/H4BG2KX8) have been submitted to the website http://www.labarchives.com/. All other supporting data are included as additional files.

## Author contributions

TN and HSG conceived and designed the experiments; MS, AK, ARR, MGM, and TN analyzed the data. TN, MS, and AK drafted the manuscript. All authors read and approved the final manuscript.

## Funding

The experiment was funded by the National Agricultural Innovation Project (NAIP, Component IV), the ICAR Network Project on Transgenics in Crop Plants (Maize Functional Genomics Component), and Computational Biology and Agricultural Bioinformatics (Agril.Edn.14(44)/2014-A&P). The funding agencies had no role in the study design, data collection and analysis, the decision to publish, or the preparation of the manuscript.

### Conflict of interest statement

The authors declare that the research was conducted in the absence of any commercial or financial relationships that could be construed as a potential conflict of interest. The reviewer DS and handling Editor declared their shared affiliation, and the handling Editor states that the process nevertheless met the standards of a fair and objective review.

## Abbreviations

GS: Genomic selection
RR: Ridge regression
LASSO: Least Absolute Shrinkage and Selection Operator
EN: Elastic Net
RF: Random Forest
RKHS: Reproducing Kernel Hilbert Space
MAS: Marker Assisted selection
QTL: Quantitative trait loci
GEBV: genomic estimated breeding value
SNP: Single nucleotide polymorphism
ASI: anthesissilking Interval
GY: Grain yield
KR: number of kernels per row
KRN: number of kernel rows
EG: Ear girth
EL: Ear length
HKW: 100 kernel weight
GWA: genome wide association
CV: cross-validation
TF: Transcription factor.
